# Medical education: past, present and future

**DOI:** 10.1007/s40037-012-0002-7

**Published:** 2012-02-07

**Authors:** Geoff Norman

**Affiliations:** Department of Clinical Epidemiology and Biostatistics, MDCL 3519, McMaster University, 1200 Main St. W., Hamilton, ON L8N3Z5 Canada

**Keywords:** Medical education, Clinical teaching, Basic science teaching

## Abstract

This article reviews changes in undergraduate and postgraduate medical education since the Flexner report of 1910. I argue that many of the changes in the twentieth century could be viewed as ‘post-Flexnerian’, and related to the integration of biomedical science in the preclinical medical curriculum. I then go on to argue that recent changes in the health care systems worldwide will force a critical re-examination of our approach to clinical education—a ‘post-Oslerian’ era. I suggest that one approach would be to decouple clinical education from clinical care, to some degree, and supplement with curricula designed around careful sequencing of simulated cases.

This is perhaps a good time in my own career, which is now winding down after 40 years, as well as an important time for the journal, to take a look back and a peek into the future. Winston Churchill said, ‘The farther backward you can look, the farther forward you are likely to see’. Keeping this in mind, I intend to take a sweeping look back over 100 years, although I am not so foolish as to cast my vision forward more than into the very immediate future. If this may be viewed as timidity on my part, I recommend a book I recently read, called ‘Future Babble,’ [1] which discusses human inadequacy at predicting the future, from geology (earthquakes) to economics (recessions) that contrasts with our unshakeable belief that we’re very good at it.

I will not be writing about the scholarly *discipline* of medical education that I have been fortunate to be a part of for 40 years (although it is more correctly a field of study populated by many disciplines); rather I will discuss the process of educating physicians, how it has changed in the past century, and how it must change in the near future. In doing so, I walk among giants, and view myself as no more than a chronicler of events.

In reviewing individuals who have had a major influence on medical education in the twentieth century, two names stand above all others: Abraham Flexner and William Osler. Perhaps this is a North American bias, and in the longer history, many Europeans have contributed. But my thesis, for better or worse, revolves around the influence of these two personalities.

Flexner (1886–1959) was an American educator who was commissioned by the Carnegie Foundation to write a review of American medical education. His recommendations had the effect of closing down many freestanding medical schools and incorporating medical schools within existing universities, where students might acquire the skills of academic inquiry and the language of biomedical science. In the longer term, this might be seen as a significant initiative toward ensuring that medicine was firmly rooted in biological science (Fig. 1).

**Fig. 1:**
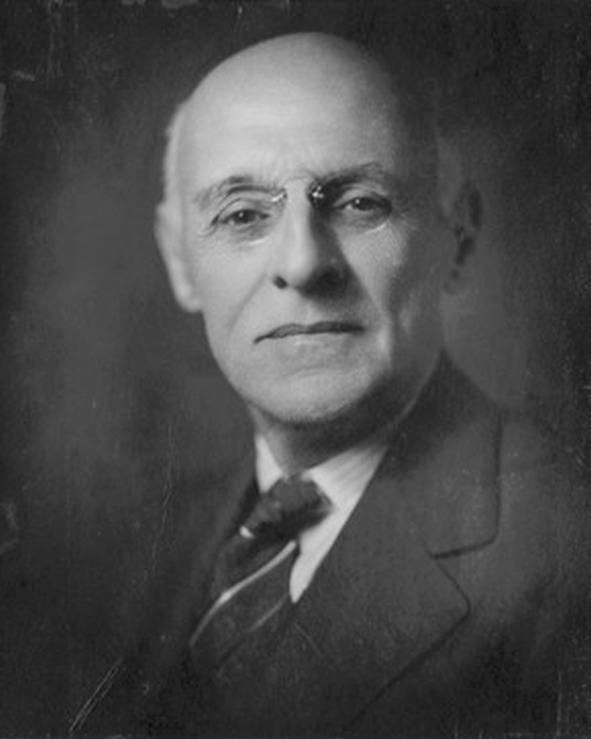
Abraham Flexner (1886–1959)

Osler (1849–1919) was a Canadian physician. Born in Bond Head, Ontario, he grew up in Dundas, now a suburb of Hamilton. His family home is about 3 km. west of McMaster University. He received an MD from McGill University, he went on to academic positions at McGill, Johns Hopkins, Pennsylvania, and Oxford. He is renowned for his approach to bedside teaching, and his insistence that students learn from their patients. One of his many quotes is, ‘He who studies medicine without books sails an uncharted sea, but he who studies medicine without patients does not go to sea at all.’ He was the ‘inventor’ of both the medical residency and the clinical clerkship (Fig. 2).

**Fig. 2:**
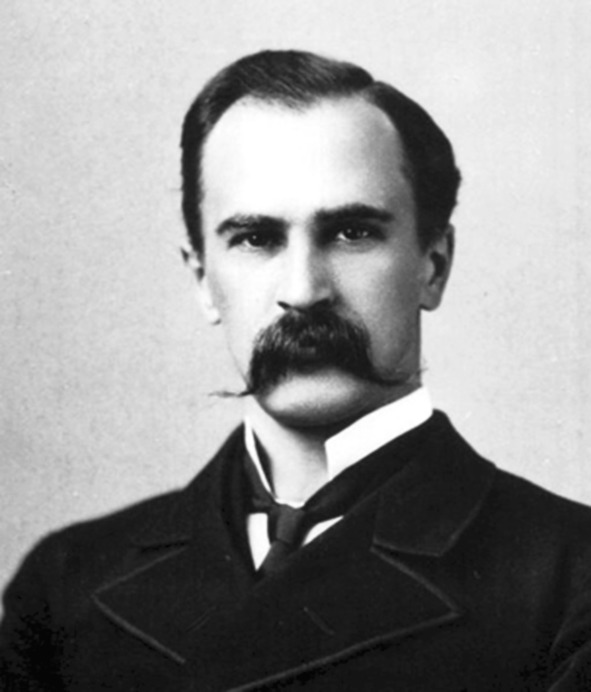
William Osler (1849–1919)

This article is not a historical review of the contributions of these two outstanding individuals; it would be pretentious for me to presume that I have the skills to write such an essay. Rather, my thesis is that the past century was strongly influenced by Flexner’s report, both directly and indirectly, and less so by Osler. But conversely, as I examine current trends, I suggest that medical education must take seriously Osler’s admonitions to learn from patients, and failure to do so will inevitably compromise the skills of tomorrow’s doctors.

## Flexner and the lessons of the past

Medicine is both an art and a science; such a thesis has been advanced many times. But the twentieth century saw the balance between science and art shift toward the former. Clearly Flexner is not solely responsible; scientific developments such as Koch’s discovery of penicillin arose in the nineteenth century. But Flexner forged a link between medical science and medical education. I am not conversant with the early changes in American medical education that resulted, nor is it particularly relevant. But the intimate link between medical research and education in the university environment resulted in large changes in education that followed rapidly from the rapid advances in medical practice such as antibiotics and vaccinations around the Second World War. Immediately after the war, the American government invested heavily in the National Institutes of Health, which provided huge funding for medical research and created numerous positions in university medical schools for basic scientists. The consequence for education was predictable—an expansion of the preclinical curriculum to ‘cover’ the many medical advances, and control of the curriculum in the hands of the basic scientists. While these changes are not a direct consequence of the Flexner report, undoubtedly Flexner would be pleased. Osler, however, may be concerned. I cannot comment on the extent to which European medical education saw similar changes; however, the extent of American hegemony and the near-universal admiration of science and technology immediately after the war make such changes very likely.

The impact on the curriculum was predictable. There was so much science to be learned that it was not unusual for students to have 40–50 scheduled lecture hours per week. Teachers were primarily research scientists, so spent little time linking concepts to clinical medicine. Basic science facts were taught in isolation and tested frequently, all without any clinical correlates. Moreover, the sciences of education and psychology were willing partners in this enterprise. Both were dominated by the behaviourist tradition, in which the student, like the rat, is a passive and motivation-free recipient of stimuli. Curricula devolved to books of objectives, decomposing all aspects of competence into long lists of behavioural elements.

Not surprisingly, some pushback occurred: in the 1950s Case Western Reserve began an Organ System curriculum in which all courses were organized around organ systems such as ‘Cardiovascular’ so that students would be learning the anatomy of the heart at the same time they were learning the physiology of the cardiovascular system. This at least forced some curriculum integration. Perhaps a more profound change was Problem Based Learning (PBL), beginning at McMaster University in the late 1960s [2]. Indeed, PBL incorporated many of the values of the 1960s—independent, self-directed learning, individual objectives, self-assessment, small-group tutorials, minimal lectures, and no examinations. The adoption of PBL by Maastricht came first, around 1973, then many more schools followed. This led to a proliferation of studies comparing outcomes of both curricula, and to a number of systematic reviews [3, 4]. By and large, outcomes are similar despite large differences in process. One recent systematic review shows that PBL students in the Netherlands have better retention and higher scores on objective tests [5]. On the other hand, a large study of 10 years of North American graduates showed a minimal effect of PBL [6].

The PBL debate appears to have calmed down in the first decade of the twenty-first century, and it seems that there is acceptance that whatever its deficiencies or benefits, outcomes of PBL schools are similar.

In terms of my present thesis, it should be recognized that all of these changes arise in the preclinical years, thus represent a continuation of the Flexnerian legacy, integrating biomedical science into medical education. Comparable interventions in the clinical years—clerkship and residency—have not occurred. There has been some examination of community-based and integrated clerkships, and again, the evidence appears to support the null hypothesis. There has been very little emphasis on the nature of the specific experiences required to achieve competence in a clinical discipline. Instead, clerkship and residency experiences remain guided and circumscribed by the nature of the patient care demands. Admittedly, there has been some attempt to facilitate acquisition of clinical skills (e.g. intubation, laparoscopy, suturing) using simulation, but these are often inadequately integrated with the rest of the curriculum.

## Osler and clinical education in the future

There is some cause for concern that the current approach to clinical education, dictated primarily by patient care demands within the health care system, is gradually eroding the quality of clinical education. Academic clinicians frequently raise the issue of restriction of resident working hours and its impact on learning. Interestingly, the acceptable maximum differs substantially between North America and Europe—48 h in the EU, and 88 h in the US. But this is only the most visible part of the problem. In my view, far more critical is the change in the nature of the clinical experience. The general medicine ward where Osler conveyed his skills at the bedside no longer exists. There are fewer and sicker patients admitted to hospital, for shorter time periods. They are older and have more multi-system disease. Data from the National Health Service (UK) comparing 2010 to 2000, a 10 year period, show:

- Shorter hospital stay for admitted patients
  7.8 to 5.6 days 2000–2010
- Reduced suitability of patients for learning
  Elderly, chronic disease, multi-system
  66% increase of admissions for patients over 75
- Reduced number of admissions overall
- More patients handled on an outpatient basis
- Reduced suitability of ward for learning
  More homogeneous, more procedure-orientation

As the supply side (patients) shrinks, the demand side (learners) increases as medical schools have responded to physician shortages by substantially increasing enrolment, and as other health professional programmes such as physician assistant programmes are initiated. One response to these pressures is to seek out additional clinical sites outside the academic environment, in rural or other community sites. While this may increase access of learners, it creates additional problems in the increased use of non-academic clinicians, and the difficulty of accessing educational materials from remote sites (leading to more research on distance education and web-based learning).

There is no way for educators to have any impact on clinical environments. These changes are a consequence of many more converging forces than can possibly be influenced by educators. We may continue to pursue educational goals in the clinical settings, and should work, as much as possible, to adapt the clinical environment to optimise learning. But as we come to understand expertise, we also come to appreciate the increasing gap between optimal strategies to achieve expertise and the real environments in which our learners function.

I propose a radical solution. In addition to providing experience in the clinical environment, a significant amount of clinical learning, both undergraduate and postgraduate, should occur in carefully engineered simulated settings. Such a proposal is not at all infeasible; as a consequence of the digital revolution of the past decade, we have a proliferation of highly sophisticated simulations for learning everything from basic perceptual skills such as cardiac auscultation to highly complex skills such as ‘weaning’ cardiac patients from bypass. But, to a large degree, these simulations reside under green sheets in clinical skills centres and are rarely part of integrated curricula.

Second, although the cost and fidelity of these simulations is highly variable, literally from €10 to €100,000, there is a growing literature that suggests that low fidelity and low cost simulators can provide a learning experience that closely approximates gains from high fidelity simulations [7]. In addition, although simulators remain inferior to actual clinical experience, there is ample evidence that skills acquired in simulator settings can be applied (transferred) to the real setting [8]. Thus, the evidence to date is that a simulated clinical curriculum is both feasible at relatively modest cost (since expensive high fidelity simulations are rarely necessary) and educationally valid.

Finally, it may be the case that learning in a simulated setting, all other things being equal, may not be as effective as learning in an equivalent real setting with real patients. But all other things are *not* equal. My intent in advocating this approach derives from the recognition, as described above, that the real setting is far from optimal and is likely, in future, to grow worse, not better.

## Simulation and the optimal clinical curriculum

Although historically, elite performance in many domains was viewed as primarily a consequence of native talent, more recent evidence has challenged this view. Ericsson has shown in a variety of domains that it takes 10,000 h of deliberate practice to become an expert [9]. However, it is critical to take note of the adjective *deliberate*. Simply seeing a sequence of problems, without reflection or carefully engineered difficulty, is not deliberate practice. Instead, to profit from practice, the individual must deliberately move to the edge of his domain of competence, and systematically practise and receive feedback at this level of difficulty.

A second critical observation with respect to medical expertise is that these experiences are not a matter of practising. Rather, the experiences comprise a second corpus of knowledge that resides in a different area of the brain from the formal knowledge of the preclinical curriculum [10]. A recent paper [11] shows that learners’ perception of medical expertise is that it derives from a large number of case experiences that enable the expert to tailor his approach to the individual patient. This view of expertise is, I think, completely consistent with Osler’s view. One becomes an expert clinician first and foremost by learning from, and building on, patient experience.

The emphasis, then, is not on devising clinical skills teaching around specific simulators for specific skills, the usual focus of simulations. Rather, I suggest we create an environment where students can work up a series of cases that adequately represents the speciality domain. I am not particularly worried about the characteristics of the individual simulation; there is ample evidence from systematic reviews that various formats lead to equivalent learning [12–14]. Rather I am interested in assembling a large number of cases that ultimately exemplify all the diagnostic, management and motor skills required for expertise in the domain. There have been some attempts along these lines already. The UMedic paediatric problems, 28 in number, are used by about 80% of North American medical schools to ensure that all clinical clerks ‘see’ all the important clinical problems in paediatrics. The ‘clinical presentations’ curriculum at Calgary [15] has created a total of 129 clinical presentations that are claimed to encompass all important medical problems.

However, expertise does not arise from an extensive and systematic workup of a single case; as I discussed earlier, our current understanding of expertise is that it derives from both a formal systematic knowledge base and extensive experience with many variants of cases. It might be argued that we run a real risk of distorting diagnostic reasoning by introducing students to a large series of simulated cases which have been deliberately sampled for educational relevance, not prevalence. Perhaps, but most health care settings, particularly in-patient, deal with an unrepresentative sample of cases, as pointed out by White many years ago [16]. In any case, while students must learn that most headaches are tension headaches, not brain tumours, the base rate is only one piece of evidence in arriving at a diagnostic conclusion for a patient with a headache. A simulated environment may well provide a far more optimal situation than the current real world for sharpening diagnostic skills.

But is that all there is? In constructing our simulated health sciences centre, is it simply a matter of having lots and lots of cases available, sampled according to some blueprint, in a variety of formats? If that was all there was, then we would have difficulty in providing sufficient clinical experience within the 48 h/week allocated. Moreover, evidence from aviation suggests that even the best simulation has an efficiency of about 0.5 compared with real experiences (2 h in simulator = 1 h of the real thing). We must do something to increase the efficiency of clinical learning.

The key is to recognize the importance of sequencing. A real clinical setting is a very inefficient place to learn diagnosis. Common things are common, and it is very difficult to arrange experiences so that students learn to differentiate between common benign conditions and the rarer, confusable, ‘don’t miss’ diagnoses. Moreover, the sequence of presentations is determined by the waiting room, not the curriculum, so that two cases of chest pain with confusable diagnoses, an ideal learning situation, may arise weeks or months apart, or never.

Intuitively, an ideal situation for learning diagnosis would be to see a series of cases side by side where two factors are engineered—different presentations of the same diagnosis or condition and similar presentations of different diagnoses. In this manner, the clinician would learn those features or aspects of the case that discriminate among different conditions. Acquisition of perceptual and motor skills could also be enhanced by practice on multiple cases from typical to atypical. Fortunately, cognitive psychology provides evidence for specific approaches that can increase efficiency of learning. Mixed practice, wherein examples from confusable cases are practised and diagnosed together, has shown increases in efficiency of the order of 50% over blocked practice [17], where one sees examples of one condition, then examples of the next, and so on. Distributed practice, where learning is deliberately spread out over several sessions, can result in learning gains compared with massed practice, where all learning occurs in one session [18].

## Conclusions

As the twenty-first century unfolds, the clinical education of medical students is under constant erosion as a consequence of demographic changes in the population they serve and economic and organizational changes in the health care system designed to respond to these pressures. The consequence is that students cannot expect the same kind of extensive and comprehensive experience with patients that may have been accepted as ordinary a century ago. To cope with these changes will require extensive and imaginative changes in medical education. The solution I have suggested involves decoupling clinical education from clinical service, then carefully engineering education to optimize these experiences.

## Essentials

- In the twentieth century, innovations in medical education were primarily confined to the preclinical curriculum.
- Health care systems worldwide are under increasing pressures that together result in less than optimal educational experiences.
- Medical education in the twenty-first century must come to grips with these changes.
- One possible solution is to implement a parallel curriculum designed around careful sequences of clinical cases using emerging digital technologies.
